# Supplementary material for: Universal scaling relations for the rational design of molecular water oxidation catalysts with near-zero overpotential
Source: Nat Commun. 2019 Nov 8;10:4993. doi: 10.1038/s41467-019-12994-w (PMC6841662; doi:10.1038/s41467-019-12994-w)
Supplement: Supplementary file 1 — Supplementary Information [file 41467_2019_12994_MOESM1_ESM.pdf]

Supplementary Figures

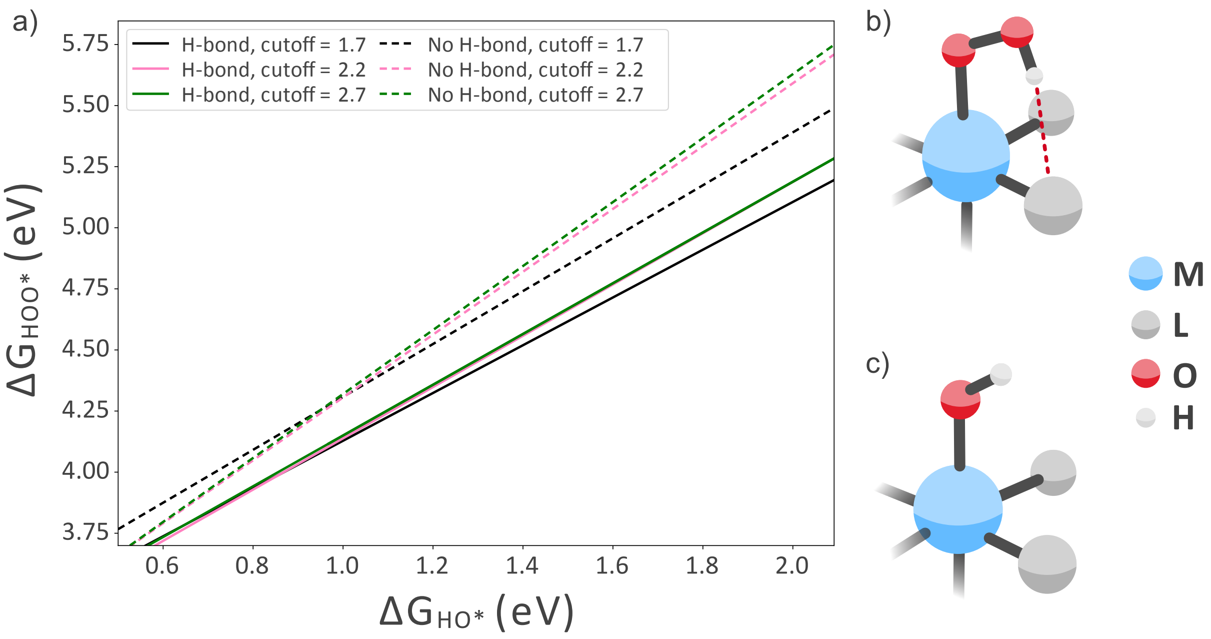

Supplementary Figure 1. (a) Linear scaling relation between the HO* and HOO* intermediates for the molecular OER catalysts investigated in this work with (solid lines) and without (dashed lines) for varying cutoffs specifying the maximum distance of a H-bond distance. Further illustrations of b) the H-bond which can occur from the HOO* fragment and c) the HO* intermediate, where H-bonds are less likely to form.

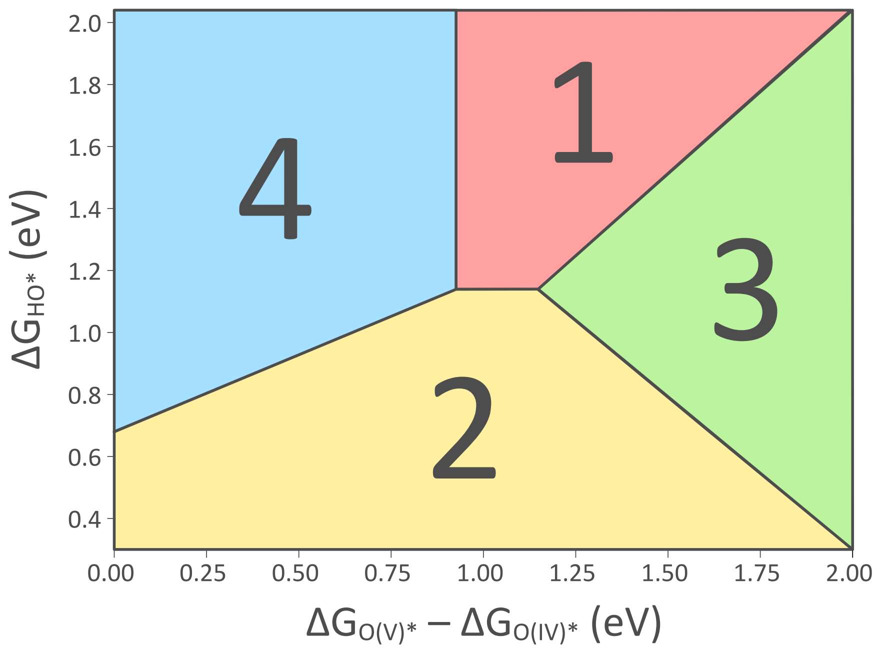

Supplementary Figure 2. 3D volcano plot using the novel OER descriptor, $\Delta G_{O(V)*} - \Delta G_{O(IV)*}$. The different colored regions represent different areas in which a specific step is likely to be the potential limiting step. A detailed derivation of the assignment of these areas is provided in the Supplementary Note 1.

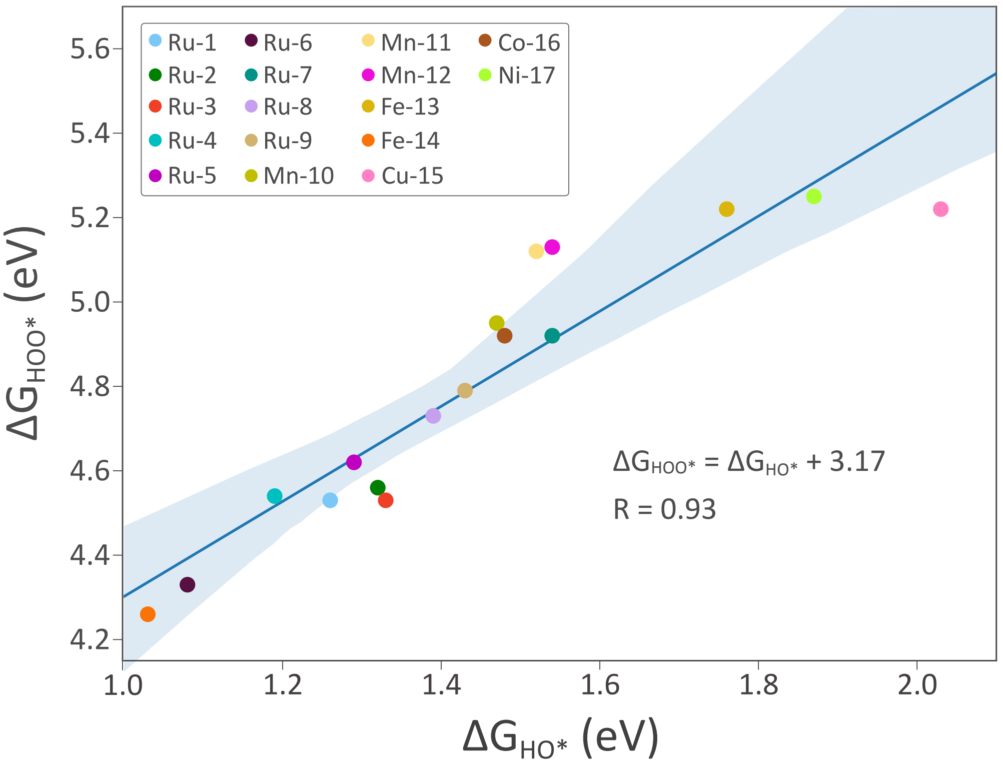

Supplementary Figure 3. OER scaling plot without dispersion corrections. The overall effect of adding dispersion corrections is to stabilize the HO* and HOO* intermediates relative to the intermediate with a vacancy, but the main conclusions of our work are robust regardless of this subtle choice of method.

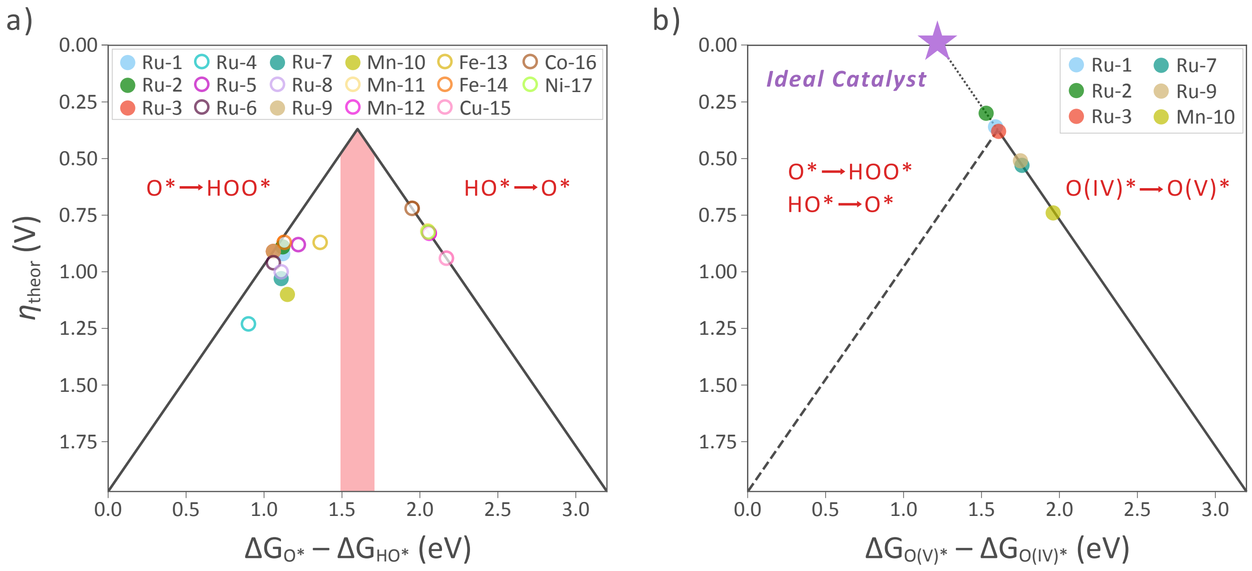

Supplementary Figure 4. Volcano plots without dispersion correction corrections for (a) the conventional OER descriptor and (b) the one-electron oxidation descriptor. We note a general stabilization of the HO* with respect to the O* intermediate, when compared to Figure 4, the data with dispersion. This leads to a reduction or increase in the predicted overpotential for catalysts on the left or right of the volcano, respectively. In any case, the most active catalysts remain some distance away from the top of the volcano irrespective of the addition of dispersion corrections.

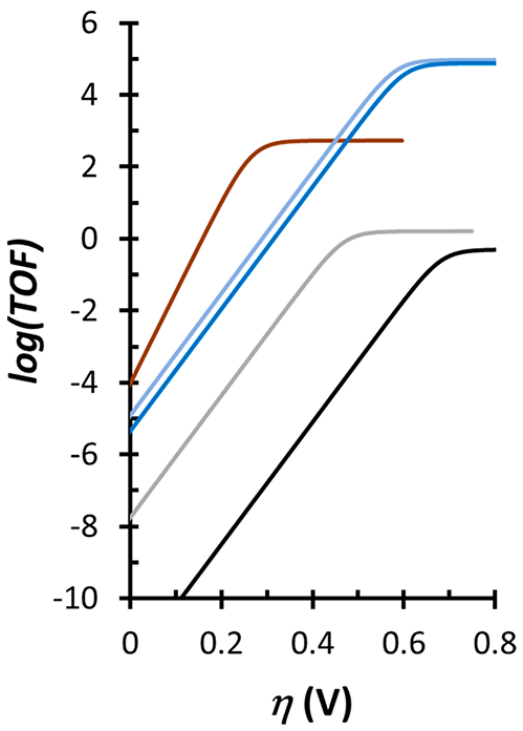

Supplementary Figure 5. Data from electrochemical experiments on a variety of molecular OER complexes. The dark blue and brown lines represent **Ru-7** and **Ru-3**, respectively. Figure adapted with permission from Ref. [62] in the main text. Copyright 2019 American Chemical Society. The predicted overpotentials for the most active OER catalysts, including **Ru-7** and **Ru-3**, is discussed in the Supplementary Note 2 in the context of their reported experimental TOFs.

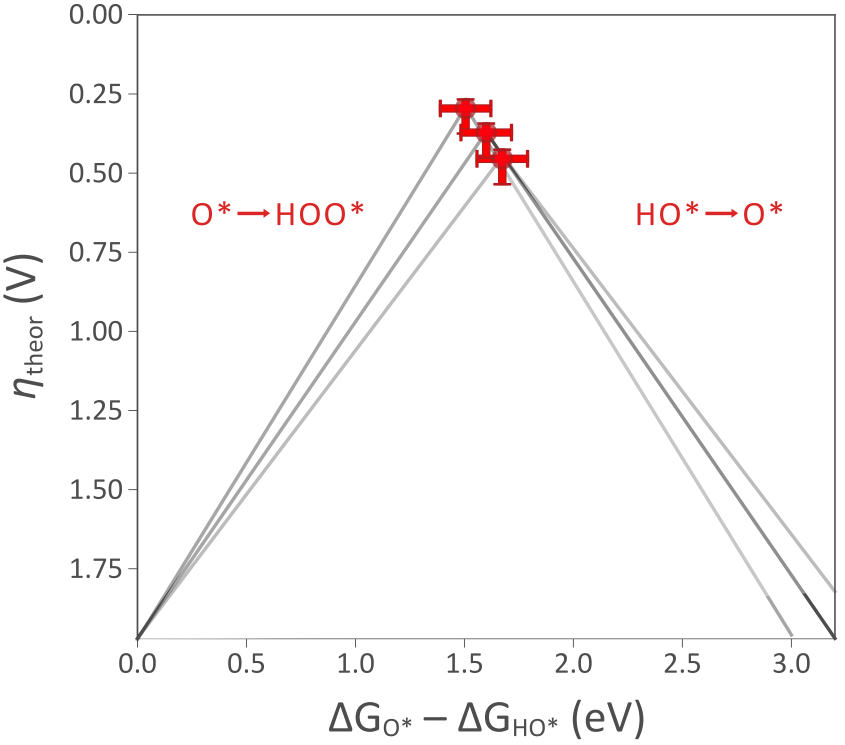

Supplementary Figure 6. Illustration of the effect of both the uncertainty in the overpotential wall, shown by the three distinct OER volcano plots, and the effect of the DFT uncertainty from the OER descriptor, $(\Delta G_{O*} - \Delta G_{HO*})$, on the calculated theoretical overpotential. For a detailed discussion on this topic, see Supplementary Note 3.

Supplementary Tables

Supplementary Table 1. Calculated and experimental reduction potentials ($E_{theor.}$ and $E_{exp.}$, respectively), in V vs RHE, using different DFT functionals. The mean absolute error (MAE) for each catalyst is provided. The lowest errors obtained for each catalyst are highlighted in bold.

Catalyst	Redox Pair	Functional	$E_{theor.}$	$E_{exp.}$	MAE
Ru-2	$[Ru(III){-}OH_2]^{+}$ / $[Ru(II){-}OH_2]$	B3LYP	0.63	0.43	**0.20**
		TPSSh	0.64		0.21
		BP86	0.80		0.37
Ru-9	$[Ru(III)OOH]^{2+}$ / $[Ru(II)OOH]^{+}$	B3LYP	–0.07	0.30	0.30
		TPSSh	–0.15		0.45
		BP86	0.02		**0.28**
Mn-12	$[Mn(IV)Mn(IV)]^{4+}$ / $[Mn(III)Mn(IV)]^{3+}$	B3LYP	1.87	1.24	0.63
		TPSSh	1.32		**0.08**
		BP86	6.43		5.19
Co-16	$[Co(III){-}OH]^{2+}$ / $[Co(II){-}OH_2]^{2+}$	B3LYP	1.53	0.75	0.78
		TPSSh	1.22		0.47
		BP86	0.32		**0.43**

Supplementary Table 2. Computed relative Gibbs energies (in eV) and theoretical overpotentials (in V) for catalysts which do and do not undergo an extra oxidation step. Catalysts which have a value of $\Delta G_{3'}$ and positive accompanying $\Delta G_{4'}$ undergo this extra oxidation step.

**Catalyst**	ΔG_1	ΔG_2	$\Delta G_{3'}$	$\Delta G_{3/4'}$	$\Delta G_{4/5'}$	η
**Ru-1**	1.02	1.17	1.62	0.36	0.74	0.39
**Ru-2**	1.09	1.16	1.52	0.48	0.67	0.29
**Ru-3**	1.13	1.11	1.60	0.37	0.71	0.38
**Ru-4**	1.03	0.95	–	2.36	0.58	1.13
**Ru-5**	1.14	1.28	–	2.01	0.49	0.78
**Ru-6**	0.89	1.13	2.32	–0.25	0.83	0.84
**Ru-7**	1.54	1.20	1.74	0.30	0.14	0.51
**Ru-8**	1.20	1.16	–	2.05	0.51	0.82
**Ru-9**	1.14	1.14	1.79	0.16	0.69	0.56
**Mn-10**	1.32	1.11	1.96	0.37	0.16	0.73
**Mn-11**	1.11	2.11	–	1.40	0.30	0.88
**Mn-12**	1.24	2.12	–	1.39	0.17	0.89
**Fe-13**	1.56	1.43	–	1.98	-0.05	0.75
**Fe-14**	0.62	1.21	2.20	–0.22	1.11	0.75
**Cu-15**	2.04	2.21	–	0.90	–0.23	0.98
**Co-16**	1.13	2.06	–	1.29	0.44	0.83
**Ni-17**	1.71	2.11	–	1.14	-0.04	0.88

Supplementary Table 3. Computed absolute and relative (*i.e.* binding energies, BE) electronic and Gibbs energies of all the intermediates for each modelled catalyst.

Catalyst	Species	Multiplicity	E (Ha)	ΔE_{BE} (eV)	G (Ha)	ΔG_{BE} (eV)
**Ru-1**	Ru(II)–*	1	–1769.461435	–	–1769.085071	–
	Ru(III)–OH	2	–1845.285084	0.77	–1844.898797	1.02
	Ru(IV)=O	3	–1844.642247	2.28	–1844.267613	2.20
	Ru(V)=O	4	–1844.427527	3.84	–1844.050900	3.81
	Ru(III)–OOH[a]	2	–1920.436978	3.83	–1920.046188	4.18
	Ru(IV)–dimer	5	– 3688.917407	–1.70[c]	–3688.136025	–0.93[c]
**Ru-2**	Ru(II)–*	1	–1801.484622	–	–1801.131987	–
	Ru(III)–OH	2	–1877.306099	0.83	–1876.943318	1.09
	Ru(IV)=O	3	–1876.665632	2.27	–1876.312676	2.25
	Ru(V)=O	4	–1876.451563	3.82	–1876.099556	3.77
	Ru(III)–OOH[a]	2	–1952.456158	3.94	–1952.090619	4.24
	Ru(IV)–dimer[c]	5	-3752.950094	-1.28	-3752.219888	-0.57
**Ru-3**	Ru(II)–*	1	–1540.781359	–	–1540.443174	–
	Ru(III)–OH	2	–1616.602794	0.83	–1616.252867	1.13
	Ru(IV)=O	3	–1615.961807	2.29	–1615.623775	2.25
	Ru(V)=O	4	–1615.745473	3.89	–1615.407574	3.85
	Ru(III)–OOH[a]	2	–1691.755202	3.88	–1691.402521	4.22
	Ru(IV)–dimer[c]	5	-3231.533954	-1.17	-3230.825323	-0.28
**Ru-4**	Ru(III)–*	2	–1429.417374	–	–1429.057701	–
	Ru(IV)–OH	3	–1505.241689	0.75	–1504.871147	1.03
	Ru(V)=O	4	–1504.606285	2.06	–1504.248235	1.98
	Ru(IV)–OOH[b]	3	–1580.386629	4.01	–1580.012891	4.34
**Ru-5**	Ru(III)–*	2	–1542.817136	–	–1542.447151	–
	Ru(IV)–OH	3	–1618.637760	0.85	–1618.256518	1.14

	Ru(V)=O	4	–1617.991907	2.44	–1617.621468	2.42
	Ru(IV)–OOH[b]	3	–1693.781349	4.14	–1693.399046	4.43
**Ru-6**	Ru(II)–*	1	–1363.863714	–	–1363.548697	–
	Ru(III)–OH	2	–1439.693636	0.60	–1439.367219	0.89
	Ru(IV)=O	3	–1439.053015	2.05	–1438.737518	2.02
	Ru(V)=O	4	–1438.810387	4.37	–1438.494919	4.35
	Ru(III)–OOH[b]	2	–1514.840162	3.81	–1514.512764	4.10
**Ru-7**	Ru(II)–*	1	–1709.277448	–	–1708.925402	–
	Ru(III)–OH	2	–1785.082706	1.27	–1784.720222	1.54
	Ru(IV)=O	3	–1784.438723	2.81	–1784.088075	2.74
	Ru(V)=O	4	–1784.218715	4.52	–1783.866870	4.48
	Ru(III)–OOH[a]	2	–1860.231358	4.42	–1859.864521	4.78
**Ru-8**	Ru(III)–*	3	–2320.960846	–	–2320.362349	–
	Ru(IV)–OH	4	–2396.780644	0.88	–2396.169509	1.20
	Ru(V)=O	5	–2396.138572	2.36	–2395.538655	2.37
	Ru(IV)–OOH[a]	4	–2471.928043	4.06	–2471.314760	4.41
**Ru-9**	Ru(II)–*	1	–1908.379427	–	–1907.803352	–
	Ru(III)–OH	2	–1984.200355	0.85	–1983.612678	1.14
	Ru(IV)=O	3	–1983.557116	2.36	–1982.982577	2.29
	Ru(V)=O	4	–1983.333219	4.17	–1982.759453	4.08
	Ru(III)–OOH[a]	2	–2059.351050	3.94	–2058.762007	4.24
**Mn-10**	Mn(II)–*	6	–1266.810381	–	–1266.463762	–
	Mn(III)–OH	5	–1342.626660	0.97	–1342.266550	1.32
	Mn(IV)=O	4	–1341.992859	2.23	–1341.637630	2.43
	Mn(V)=O	3	–1341.763936	4.18	–1341.408605	4.38
	Mn(III)–OOH[b]	5	–1417.767263	4.34	–1417.404290	4.74
**Mn-11**	Mn(III)–*	5	–1227.081183	–	–1226.754556	–
	Mn(IV)–OH	4	–1302.906290	0.73	–1302.565160	1.11
	Mn(V)=O	3	–1302.230586	3.13	–1301.899382	3.22
	Mn(IV)–OOH[b]	4	–1378.042881	4.21	–1377.699278	4.62
**Mn-12**	Mn(III)–*	8	–1919.706849	–	–1919.278593	–

	Mn(IV)–OH	1	–1995.528760	0.82	–1995.084332	1.24
	Mn(V)=O	6	–1994.849611	3.31	–1994.418420	3.36
	Mn(IV)–OOH[b]	7	–2070.664133	4.33	–2070.218666	4.75
**Fe-13**	Fe(III)–*	4	–2420.099130	–	–2419.858797	–
	Fe(IV)–OH	3	–2495.905602	1.24	–2495.652945	1.56
	Fe(V)=O	4	–2495.251392	3.05	–2495.012270	2.99
	Fe(IV)–OOH[b]	3	–2571.044661	4.65	–2570.790935	4.97
**Fe-14**	Fe(II)–*	3	–1041.772009	–	–1041.428137	–
	Fe(III)–OH	2	–1117.619785	0.11	–1117.256564	0.62
	Fe(IV)=O	3	–1116.976084	1.64	–1116.623900	1.84
	Fe(V)=O	4	–1116.737215	3.86	–1116.385804	4.03
	Fe(III)–OOH[a]	2	–1192.766970	3.31	–1192.402658	3.81
**Cu-15**	Cu(II)–*	2	–1353.609050	–	–1353.348108	–
	Cu(III)–OH	1	–1429.398080	1.71	–1429.124425	2.04
	Cu(IV)=O	2	–1428.714801	4.32	–1428.454907	4.26
	Cu(III)–OOH[a]	1	–1504.549031	4.80	–1504.273294	5.15
**Co-16**	Co(II)–*	4	–1689.425377	–	–1688.989311	–
	Co(III)–OH	1	–1765.250084	0.74	–1764.799248	1.13
	Co(IV)=O	2	–1764.572417	3.20	–1764.135313	3.19
	Co(III)–OOH[b]	1	–1840.392146	4.07	–1839.939301	4.48
**Ni-17**	Ni(II)–*	1	–2305.501674	–	–2304.861453	–
	Ni(III)–OH	2	–2381.290337	1.72	–2380.650122	1.71
	Ni(IV)=O	3	–2380.614802	4.12	–2379.984367	3.82
	Ni(III)–OOH[a]	2	–2456.443085	4.76	–2455.793706	4.96

[a] Intramolecular H–bond present.

[b] Intramolecular H–bond not present.

[c] Energy change calculated with respect to the Ru(V)=O energy.

Supplementary Table 4. Experimental conditions and TOFs reported for the most active Ru catalysts investigated in this work. In all the cases, cerium ammonium nitrate (CAN) was employed as chemical oxidant. We note that comparing DFT predicted overpotentials to the experimentally observed catalytic activity is challenging since the conditions applied to measure performance are not uniform across all investigations, as seen below. However, upon comparing our results with the reported experimental TOFs (see Supplementary Note 2), we feel confident that our calculations predict the right trend.

Catalyst	TOF (s^{-1})	pH	[Catalyst]	Chemical Oxidant	Reference
**Ru-1**	303	1	2.16×10^{-4} M	CAN (0.48 M)	29
**Ru-2**	286	1	2.22×10^{-4} M	CAN (0.365 M)	30
**Ru-3**	32	1	2.16×10^{-4} M	CAN (0.48 M)	29
**Ru-7**	8,000	7	1.7×10^{-3} M	CAN (n.a.)	31
**Ru-7**	50,000	10	1.7×10^{-3} M	CAN (n.a.)	31

Supplementary Notes

Supplementary Note 1

The shape of the 3D volcano plot using the novel OER descriptor, $\Delta G_{O(V)*} - \Delta G_{O(IV)*}$, depicted in Figure 5 and Supplementary Figure 2 can be explained by considering the equations that determine the boundaries separating distinct regions which denote the potential limiting step (PLS).

To begin the analysis, we define the Gibbs binding energies of the different OER intermediates within the computational hydrogen electrode model as follows:

$$\Delta G_{HO^*} = G_{HO^*} + 0.5 * G_{H_{2(g)}} - G_* - G_{H_2O_{(l)}}$$

$$\Delta G_{O^*} = \Delta G_{O(IV)^*} = G_{O^*} + G_{H_{2(g)}} - G_* - G_{H_2O_{(l)}}$$

$$\Delta G_{HOO^*} = G_{HOO^*} + 1.5 * G_{H_{2(g)}} - G_* - 2 * G_{H_2O_{(l)}}$$

$$\Delta G_{O(V)^*} = G_{O(V)^*} + G_{H_{2(g)}} - G_* - G_{H_2O_{(l)}} + eE^*_{abs}$$

Where, E^*_{abs} is the value assigned to the normal hydrogen electrode potential in water, for which we used a previously calculated value of –4.28 V. The method applied to arrive at this value uses the absolute solvation free energy of the proton, which is calculated using the cluster pair approximation method, enthalpy and entropy corrections are then added by numerically solving equations from Fermi–Dirac statistics. This method was shown to give consistent and sufficient agreement with experimental data, as reported in Ref. [55] of the main text.

We then use the above binding energies to determine the PLS using the established linear scaling relationship for molecular catalysts, shown in Figure 3, and represent the values on the *x* and *y* axes as *x* and *y*, respectively.

$$\Delta G_1 = \Delta G_{HO*} = y$$

$$\Delta G_2 = \Delta G_{O(IV)*} - \Delta G_{HO*} = \Delta G_{O(IV)*} - y$$

$\Delta G_{3'} = \Delta G_{O(V)*} - \Delta G_{O(IV)*} = x$

$\Delta G_{4'} = \Delta G_{HOO*} - \Delta G_{O(V)*} = (3.2 + \Delta G_{HO*}) - \Delta G_{O(V)*} = 3.2 + y - x - \Delta G_{O(IV)*}$

$\Delta G_{5'} = 4.92 - \Delta G_{HOO*} = 4.92 - (3.2 + \Delta G_{HO*}) = 1.72 - \Delta G_{HO*} = 1.72 - y$

Using simple algebra, we can determine the expected shape of the intersections between the distinct regions shown in the 3D volcano representation in Supplementary Figure 2.

$Step\ 1 = Step\ 2$

$y = 0.5(\Delta G_{O(IV)*})$

$Step\ 1 = Step\ 3'$

$y = x$

$Step\ 1 = Step\ 4'$

$x = 3.2 - \Delta G_{O(IV)*}$

$Step\ 2 = Step\ 3'$

$y = -x + \Delta G_{O(IV)*}$

$Step\ 2 = Step\ 4'$

$y = 0.5x + \Delta G_{O(IV)*} - 1.6$

Step 5' is not seen on the volcano plot in Supplementary Figure 2 because it is not limiting until ΔG_{HO*} is sufficiently negative. It should also be noted that there is an important distinction between the two volcano plots using the conventional and new OER descriptors shown in Figure 5. In particular, the overpotential can be directly obtained from the conventional volcano plot by computing the ΔG_{HO*} and ΔG_{O*} values – assuming that the first and last OER steps are not PLS, which is generally the case. On the contrary, the volcano plot using the novel OER descriptor requires three values, ΔG_{HO*}, $\Delta G_{O(V)*}$ and $\Delta G_{O(IV)*}$ (as can be seen above for $\Delta G_{1-5'}$).

Supplementary Note 2

Notably, the catalyst we predict to exhibit the lowest theoretical overpotential is **Ru-2**, followed by **Ru-1** and **Ru-3**. In experiments, this trend is respected also, with the **Ru-1** and **Ru-2** catalysts showing similar yet undoubtedly superior performance than **Ru-3**. The reason for the apparent difference between the experimental TOFs reported for **Ru-3** and **Ru-1-2** and our theoretical overpotentials may be ascribed to the fact that these Ru catalysts have been experimentally proposed to follow a I2M mechanism rather than the WNA assumed in our theoretical overpotentials. On the other hand, **Ru-7** shows remarkable activity at high pH, as seen in Supplementary Table 4. However, to the best of our knowledge, this catalyst shows no activity in acidic media under which the other catalysts were tested. Furthermore, when **Ru-3** is tested at pH = 7 (Supplementary Figure 5), it displays a better activity at low potentials than **Ru-7**, which is concurrent with our calculations predicting a lower overpotential for **Ru-3**. Hence, the predictive (both qualitatively and semi-quantitatively) strength of our approach is further demonstrated.

Supplementary Note 3

In the review process of this paper, one referee suggested a more detailed explanation about the uncertainty in the overpotential wall. The source of this uncertainty is in the deviation from the line of best fit in Figure 2, and the uncertainty can be calculated as the standard deviation from the mean value of $(\Delta G_{HOO*} - \Delta G_{HO*})$. In the following we provide a generalized explanation for the uncertainty of the overpotential wall to illustrate this is more detail.

Given an uncertainty σ, and a mean $\Delta G_{HOO*} - \Delta G_{HO*}$ value of 3.2 eV, it may be tempting to assume that this uncertainty is the same as the uncertainty associated with the overpotential wall. However, we will now set out our interpretation to show that the interval of possible overpotential walls is in fact given by:

$$\left[\frac{3.2 - \sigma}{2} - 1.23, \frac{3.2 + \sigma}{2} - 1.23\right]$$

As an instructive demonstration, in Supplementary Figure 6 we have constructed three putative volcano plots assuming different constraints imposed by the three putative values of $(\Delta G_{HOO*} - \Delta G_{HO*})$, given by the OER scaling relations. The *y*-axis is the theoretical overpotential, which is indirectly calculated using DFT according to Eq. 7 in the main text, while the *x*-axis is the so-called OER descriptor, $(\Delta G_{O*} - \Delta G_{HO*})$. Because this latter energy is calculated by DFT, it has an associated uncertainty. Importantly, this uncertainty is the same for all the points; however, due to the constraint imposed by the $(\Delta G_{HOO*} - \Delta G_{HO*})$ value obtained from the scaling, any shift away from the peak of a given volcano (located at $[\Delta G_{HOO*} - \Delta G_{HO*}]/2$ on the *x*-axis) can only increase the theoretical overpotential. These uncertainties, represented by the red error bars in Supplementary Figure 6, are distinct from σ, which is the scale of the difference between the maximum and minimum heights of the overpotential wall (given by the expressions above), also

represented in Supplementary Figure 6. Hence, the uncertainty associated with the overpotential wall is simply given by $[\sigma/2]$.
